# Agriculture modifies the seasonal decline of breeding success in a tropical wild bird population

**DOI:** 10.1111/1365-2664.12310

**Published:** 2014-07-25

**Authors:** Samantha J. Cartwright, Malcolm A. C. Nicoll, Carl G. Jones, Vikash Tatayah, Ken Norris

**Affiliations:** ^1^ Centre for Agri‐Environmental Research School of Agriculture, Policy and Development University of Reading Reading RG6 6AR UK; ^2^ Durrell Wildlife Conservation Trust Les Augrès Manor Trinity Jersey JE3 5BP Channel Islands, UK; ^3^ Mauritian Wildlife Foundation Grannum Road Vacoas Mauritius

**Keywords:** anthropogenic habitat, coupled effect, model system, phenology, prey, raptor, reintroduced, reproduction, spatio‐temporal synergy

## Abstract

1. Habitat conversion for agriculture is a major driver of biodiversity loss, but our understanding of the demographic processes involved remains poor. We typically investigate the impacts of agriculture in isolation even though populations are likely to experience multiple, concurrent changes in the environment (e.g. land and climate change). Drivers of environmental change may interact to affect demography, but the mechanisms have yet to be explored fully in wild populations.
2. Here, we investigate the mechanisms linking agricultural land use with breeding success using long‐term data for the formerly Critically Endangered Mauritius kestrel *Falco punctatus,* a tropical forest specialist that also occupies agricultural habitats. We specifically focused on the relationship between breeding success, agriculture and the timing of breeding because the latter is sensitive to changes in climatic conditions (spring rainfall) and enables us to explore the interactive effects of different (land and climate) drivers of environmental change.
3. Breeding success, measured as egg survival to fledging, declines seasonally in this population, but we found that the rate of this decline became increasingly rapid as the area of agriculture around a nest site increased. If the relationship between breeding success and agriculture was used in isolation to estimate the demographic impact of agriculture, it would significantly under‐estimate breeding success in dry (early) springs and over‐estimate breeding success in wet (late) springs.
4. Analysis of prey delivered to nests suggests that the relationship between breeding success and agriculture might be due, in part, to spatial variation in the availability of native, arboreal geckos.
5. *Synthesis and applications*. Agriculture modifies the seasonal decline in breeding success in this population. As springs are becoming wetter in our study area and since the kestrels breed later in wetter springs, the impact of agriculture on breeding success will become worse over time. Our results suggest that forest restoration designed to reduce the detrimental impacts of agriculture on breeding may also help reduce the detrimental effects of breeding late due to wetter springs. Our results therefore highlight the importance of considering the interactive effects of environmental change when managing wild populations.

## Abstract 2

Agriculture modifies the seasonal decline in breeding success in this population. As springs are becoming wetter in our study area and since the kestrels breed later in wetter springs, the impact of agriculture on breeding success will become worse over time. Our results suggest that forest restoration designed to reduce the detrimental impacts of agriculture on breeding may also help reduce the detrimental effects of breeding late due to wetter springs. Our results therefore highlight the importance of considering the interactive effects of environmental change when managing wild populations.

## Introduction

One of the most pressing research needs in ecology is to understand organisms’ responses to human‐induced habitat modification, particularly the conversion of forest habitats into agriculture, which is a major driver of biodiversity loss world‐wide. Transforming forest into agriculture involves acute structural change, by reducing the complexity of the biotic community as well as direct species replacement and the introduction of disturbance regimes that directly and indirectly impact biodiversity (Green *et al*. 2005). With global deforestation occurring at a net rate of 0·14% per annum (Food & Agriculture Organisation 2010), agricultural land predicted to expand by 10–20% by 2050 (Reid *et al*. 2005), and with evidence of declines in habitat specialist birds (Butchart *et al*. 2010), a detailed understanding of the consequences for biodiversity is imperative.

The long‐term stability of populations exposed to novel agricultural habitats depends on the impact of agriculture on demography. This is typically quantified by exploring spatial patterns in land use and performance for a discrete life‐history event, such as breeding, which is a key parameter driving variation in population growth rates (Sæther & Bakke 2000). A growing number of studies on animal populations are demonstrating habitat‐related patterns in breeding success (e.g. Kerbiriou *et al*. 2006; Amar *et al*. 2008), although they often lack the detailed ecological data needed to reveal the mechanisms involved (but see Byholm *et al*. 2007). These habitat‐related patterns can then be used to parameterize simple demographic models to explore the implications of agriculture for population stability (e.g. Wilson *et al*. 1997).

This approach views agricultural change in isolation from other drivers of environmental change. Wild populations, however, may be exposed to multiple, concurrent environmental changes (e.g. land‐use and climate change), which could act synergistically or antagonistically in terms of their impacts on population growth and stability. Failure to consider such interactive effects may result in under‐ or over‐estimates of the demographic impact of a particular driver (e.g. agriculture) when viewed in isolation. One potentially important process in this respect is the timing of breeding. The timing of breeding is important for breeding success (Perrins 1970), as it determines synchrony with environmental conditions such as food availability. However, recent studies of wild populations have shown that the timing of breeding is responding to climate change, with implications for breeding success depending on the extent to which changes in timing affects synchrony with food supplies (Visser, Holleman & Gienapp 2006; Charmantier *et al*. 2008). This means that land‐use and climate change may indirectly interact to affect breeding success, but these mechanisms have yet to be fully explored in wild populations.

Here, we aim to explore this type of interactive mechanism using an extraordinarily detailed long‐term data set on a tropical forest specialist, the Mauritius kestrel *Falco punctatus* Temminck. This population is an ideal candidate system with which to explore these ecological mechanisms because it has been intensively monitored since reintroduction to the wild and it now persists within a fragmented, degraded forest habitat surrounded by open agricultural land. This is a typical situation for many tropical forest populations, but detailed empirical data from wild populations is generally lacking (Stephens *et al*. 2004). As apex predators, raptors are particularly vulnerable to (and thus indicative of) changes in ecosystem productivity and complexity (Newton 1979; and see discussion in Sergio, Newton & Marchesi 2008), so are an ideal group with which to study the impacts of habitat change. Recent work has shown that Mauritius kestrel breeding success is reduced in agricultural habitats (Burgess *et al*. 2011), their timing of breeding is sensitive to spring rainfall patterns with early breeders generally most successful (i.e. there is a seasonal decline in breeding success; Senapathi *et al*. 2011) and spring rainfall conditions in the study area have deteriorated over time (Senapathi *et al*. 2010). The kestrel population therefore experiences spatial variation in its exposure to agriculture coupled with variation in the timing of breeding.

Specifically, we address two questions: (i) Is there any evidence that agriculture and the timing of breeding interact to affect breeding success in kestrels? and (ii) To what extent can any interactive effects be explained by differences in the prey community the kestrels exploit? We focus on breeding success because it varies spatially (Burgess *et al*. 2011) and is related to the timing of breeding. We anticipated that exposure to agriculture might reduce breeding success, at least partly because the native prey of kestrels (endemic *Phelsuma* spp. day geckos) are largely confined to forests (Vinson & Vinson 1969), whereas agricultural areas provide a mixture of native and exotic insects, reptiles, birds and mammals. Similar differences in diet have been shown to affect breeding success in other bird species (Penteriani, Gallardo & Roche 2002; Rodríguez, Johst & Bustamante 2006).

## Materials and methods

### Study System and Data

We studied an isolated population of the endemic Mauritius kestrel, which was reintroduced to the Bambous mountains, Mauritius (20·3°S, 57·7°E), in 1987, where it had previously been absent for *c*. 30 years (Jones *et al*. 1995). Since reintroduction, almost all individuals have been colour‐marked and nearly all breeding attempts monitored, resulting in spatially referenced breeding information for 675 breeding attempts and re‐sighting information for almost 900 individuals over a 22‐year period. Only a small fraction of nests fledge un‐ringed juveniles (3 in 2009), largely because these nests remain inaccessible, and by 1995, <10% of new breeders were un‐ringed (Groombridge *et al*. 2001). The breeding population numbers 49 pairs (2009/10 season) and appears to have reached carrying capacity (Butler *et al*. 2009). The only other wild population of Mauritius kestrels is over 18 km away, and there has been no documented dispersal between these two populations. Hence, the Bambous mountains population can be considered a single, closed system.

The study area spans 163 km^2^ across a mountain range and elevation varies from sea level to 626 m. The non‐agricultural habitat is a heterogeneous matrix comprising forest invaded to varying degrees by exotic trees and shrubs, including Travellers palm *Ravenala madagascariensis* Sonn. and strawberry guava *Psidium cattleianum* Sabine. Much of the area is managed for hunting, and here, the forest is interspersed with grassland pasture. The forest mosaic is bounded by vast tracts of agriculture, almost exclusively cultivated for sugar cane, but in places is devoted to banana *Musa* spp., palm *Dictyosperma album var. album* or horticulture. Occasional tree‐lined riparian corridors are the sole‐wooded link to other forest patches on Mauritius. There is therefore a clear structural dichotomy between agricultural and non‐agricultural habitats in this system.

The kestrel is territorial and monogamous with breeding pairs defending a territory around the nest site (Jones 1987). During breeding, the male provides all of the food for the pair and is the primary provider for the offspring. Nests are located in boxes or natural cavities in cliffs and trees (ratio 6:3:1). The earliest eggs are laid at the beginning of September (clutch size 2–4), hatching occurs after 28–30 days, and chicks are ringed in the nest at 12–28 days old as described in Nicoll, Jones & Norris (2006). Chicks fledge at 32–35 days (brood size 1–4) and the majority have fledged by late December, with the latest fledged by early February. Mauritius kestrels are single‐brooded but can lay second clutches if the first fails or once offspring from the first clutch fledge. Fledging coincides with the onset of the cyclone season (December to April), which varies yearly in severity (Senapathi 2009). Mauritius kestrels are independent at 3 months and can breed in their first year.

Our data set contains information for 615 first clutches during the 20‐year period 1990–2009. The number of fledglings produced by each nesting attempt was confirmed by regular nest checks in the period leading up to fledging. For each attempt, there is information on nest type, location, parent identity and the timing of laying. We excluded 65 attempts where full clutch size was unknown and a further 42 that received management intervention during nesting. This ensured only clutches with a known fate were included in the analysis and to avoid any bias in breeding success resulting from nest manipulation (Butler *et al*. 2009).

Seven canopy‐based habitat classifications were recognized from a digital map of the study area, comprising five forest classes (native, 6%; semi‐invaded, 3%; invaded, 12%; plantation, 1%; and *R. madagascariensis*, 6%), grassland (4%) and agriculture (68%) (Burgess *et al*. 2009). Habitat composition did not change appreciably during the study period. Since nests surrounded by agriculture have reduced breeding success (Burgess *et al*. 2011), we specifically calculated the proportion of agricultural habitat within a 1‐km^2^ area surrounding the nest (henceforth, simply termed ‘agriculture’). This 1‐km^2^ area is representative of a Mauritius kestrel breeding territory (Jones *et al*. 1995; Carter & Jones 1999) and has been adopted in previous work (Burgess *et al*. 2011). Daily rainfall data were available for the entire study period, and we used influential rainfall periods in the analyses (specifically, December rainfall and August rain days), following the method of Senapathi *et al*. (2011).

The data on prey delivered to the nest were based on observations during nest visits. Records comprised the prey type and the date, time, kestrel identity and location. For simplicity, we grouped prey into categories: insect, bird, mammal, agama lizard *Calotes versicolor* Daudin and native or exotic gecko. We identified gecko species as native *Phelsuma* or introduced species, since both native and introduced species co‐occur within the kestrels’ range (Cole 2005) and *Phelsuma* species can be difficult to distinguish from a distance. The prey data were collected over the same time frame as the breeding data and contained 2230 records (number of records by territory: mean = 25, range = 1–103; and by breeding season: mean = 112, range = 36–250) spanning the breeding season from 3 September to 21 February.

### Statistical Analyses

#### Agriculture, timing and breeding success

Our first objective was to identify any interactive effect between agriculture and the timing of breeding on breeding success, defined as the ratio of fledged to not‐fledged eggs per breeding attempt. Previous work showed that breeding success measured this way declines as agriculture within a breeding territory increases (Burgess *et al*. 2011), and declines as the season progresses (Senapathi *et al*. 2011), but that the timing of breeding is not related to the area of agriculture within the breeding territory (Burgess *et al*. 2011). We used generalized linear mixed models (GLMM) to model breeding success, including the female parent's identity as a random effect to account for parent‐level replication of conditions. We compared models with alternative random effect structures to ensure this did not affect our results and for brevity report these in the Supporting Information (Tables S1–S3, Supporting information). We modelled breeding success as a two‐vector matrix response, with binomial errors and a logit link. Variables/interaction terms of interest in all analyses were tested for significance using likelihood ratio tests (LRT; chi‐squared test statistic).

We initially specified a simple model with only the interaction between agriculture and the timing of breeding as fixed effects. We measured timing of breeding as the absolute date on which the first egg was laid (‘first egg date’; 1 = 1 September) rather than relative to the population‐mean timing of breeding each season (e.g. Tinbergen & Daan 1990) for two reasons. First, there is limited between‐year variation in timing of breeding in relation to the frequency of spring rainfall (Fig. S1, Supporting information). This means that relative and absolute timings are highly correlated in this system (Pearson product‐moment correlation across all individuals and years: *r*_352_ = 0·911, *P *<* *0·001). Secondly, the timing of breeding and breeding success in relation to timing are affected by independent climate processes; timing is related to the frequency of spring rainfall, whereas breeding success is related to the quantity of rainfall during the chick period in December (Senapathi *et al*. 2011). Therefore, absolute rather than relative date is a more relevant ecological measure of the timing of breeding in this system. In this initial model, we tested the significance of the interaction between timing of breeding and agriculture as well as the main effects of both variables.

We then constructed a complex model based on *a priori* decisions of potentially important ecological variables (see Burnham & Anderson 2002). This was to account for variation in breeding success attributable to other factors and to determine whether any interactive effect of agriculture and timing was explained by other correlated variables. Previous work on this population showed that breeding success is influenced by both inter‐annual differences in December rainfall (Nicoll 2004) and an interaction between the timing of breeding and December rainfall (Senapathi *et al*. 2011), so we included this interaction in the model. Parent breeding experience can affect breeding success (Forslund & Pärt 1995), and in this population, the male parent's breeding experience is important (Nicoll 2004; Burgess *et al*. 2008), so we included the number of years of male breeding experience. We included a two‐level factor identifying the type of nest cavity (box or natural cliff/tree cavity) to account for any effects of nest characteristics on breeding success. We also included clutch size to account for potential sibling competition and the number of breeding pairs within a 1‐km^2^ area around the nest to capture any competition effect from other breeding pairs. The agriculture and timing interaction was added to this complex model to assess its effect on breeding success in addition to these background variables. If it was significant, this would indicate that agriculture has a timing‐dependent effect on breeding success, independently of other significant effects.

To check whether any relationship between agriculture, timing of breeding and breeding success was affected by breeding attempts that produced no fledglings, we repeated our analyses in two further ways. First, we examined nest‐scale success, defined as a binary response with breeding attempts marked as successful (produced at least one fledgling) or failed (produced no fledglings). Secondly, we excluded all attempts which failed and repeated our original analysis of breeding success. These additional analyses are reported in Tables S2 and S3 (Supporting information).

In order to expose any bias in predicted breeding success when agriculture is considered apart from timing, we compared the predicted breeding success with varying agriculture for dry and wet springs. We first predicted the population‐mean timing of breeding for the wettest and driest recorded springs using a simple GLMM containing female identity as a random effect and August rain days as the explanatory variable (Fig. S1, Supporting information). For these mean first egg dates, we estimated breeding success with varying agriculture using the complex model described above. We then calculated the overall population‐mean first egg date from the breeding data and predicted the associated breeding success with varying agriculture in the same way and used this as a baseline. We then measured the difference between the dry and wet spring breeding success vs. the baseline breeding success for the range of agriculture coverage. Any deviation from the baseline represents the difference between ignoring or accounting for the interaction between agriculture and timing under wet or dry spring scenarios.

#### Habitat and prey

Our second main objective was to assess the extent to which any effect of agriculture on breeding success could be explained by changes in the type of prey delivered to nests. The majority of prey deliveries were geckos (gecko 78%; agama lizard 12%; bird, e.g. exotic and endemic adults and pulli, 7%; mammal, e.g. Asian musk shrew *Suncus murinus* Linnaeus, 1%; insect 0·8%; *n* = 2230; no. of kestrels = 268; no. of sites = 89). Since most prey were geckos and >95% of these were *Phelsuma* spp., prey items were simply categorized as gecko or non‐gecko. We analysed the relative probability of gecko delivery to the nest using a GLMM with a binary vector response (gecko or non‐gecko prey delivery), binomial errors and a logit link. This method treats each delivery observation as an independent trial, allowing analysis of prey delivery trends both within season and nest. We included kestrel identity as a random effect to account for pseudo‐replication when there were multiple prey deliveries by the same kestrel. Initially, we specified a univariate model with agriculture as the fixed effect and tested its significance. To determine whether any effect of agriculture remained when other conditions were accounted for, we next constructed a complex model containing other potential effects on gecko delivery. Since prey availability could be seasonal and affected by weather conditions, we included the delivery date and December rainfall in the model. Nest site elevation was included to characterize any spatial variation in prey type not related to agriculture, as field observations of Mauritius kestrel behaviour suggest that elevation could affect foraging through increases in cloud cover at higher elevations (S.J. Cartwright & M.A.C. Nicoll, personal observations). The age and sex of the kestrel delivering the prey were included to account for individual foraging experience and gender differences in prey selection. The number of breeding pairs within 1 km^2^ of the nest was included to control for competition arising from overlapping foraging areas, and the sampling season was added to account for inter‐annual variation. An interaction between agriculture and sex was included because sex‐specific differences in prey choice are common in dimorphic raptors (Newton 1979) and may be emphasized in different habitats. Before including this interaction, we checked for any gender bias in the number of delivery records in relation to agriculture using a generalized linear model with the ratio of male and female observations as a two‐vector matrix response (binomial errors and logit link) and agriculture as the explanatory variable ($\chi_{1}^{2}$ = 1·621, *P *=* *0·797; i.e. no gender bias in number of observations). We also included an interaction between agriculture and the date of prey delivery, as seasonal changes in prey may differ by habitat. All explanatory variables except sex were included as covariates. If agriculture was significant in the complex model, this would suggest that the type of prey delivered to the nest depends on the habitat within the breeding territory, independently of other variables.

#### Prey and breeding success

To investigate whether the type of prey delivered to the nest was important in linking agriculture to breeding success, we calculated a site‐specific proxy measure of gecko delivery probability (‘SGP’). This used the results of a GLMM for gecko delivery probability with kestrel identity as a random effect, sampling season as an explanatory covariate and nest site identity as a categorical effect. The parameter estimates for each nest site were extracted, so every nest site had a SGP allocated.

We then used SGP as a replacement for agriculture in the complex GLMM of breeding success. SGP was included in an interaction with the timing of breeding because it was plausible that gecko delivery has a seasonal effect on breeding success. If the interaction between SGP and the timing of breeding was significant in this model, it would suggest that the type of prey delivered to the nest is directly linked to breeding success and the effect is related to the timing of the breeding attempt.

Finally, we added agriculture and its interaction with the timing of breeding back into this complex model containing SGP. We compared the resulting model with the one that lacked agriculture. If the model containing both the agriculture and SGP terms was not significantly better at explaining variation in breeding success than the model containing SGP alone, it would suggest that SGP explains any agriculture effect on breeding success.

All models in this study were implemented in the statistical programme R (R Core Team 2014); GLMM used the lme4 package (Bates *et al*. 2014).

## Results

### Agriculture, Timing and Breeding Success

**Figure 1:**
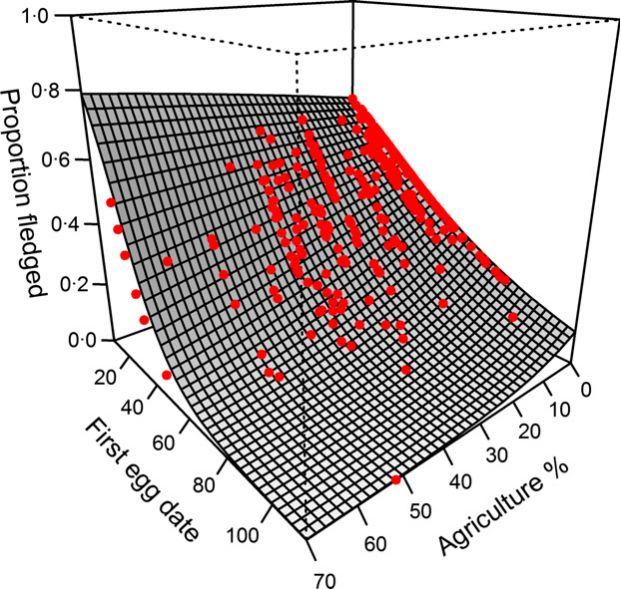
Interactive effect of timing of breeding (first egg date) and area of agriculture within the breeding territory on breeding success. Surface shows predicted trend from parameters in Table 1. Points show combinations of agriculture and timing in raw data with model predicted breeding success. First egg date scale is from 1 (1 September) to 113 (22 December). Graph made using the lattice package (Sarkar 2008).

**Figure 2:**
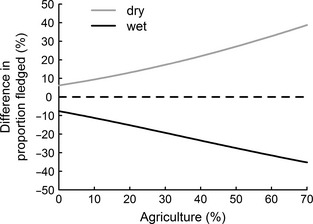
Difference in breeding success for wet and dry springs with varying agriculture extent. Solid lines indicate breeding success when the population‐mean timing of breeding for the wettest and driest springs is used, relative to the overall mean timing of breeding (dashed line). Wettest springs had 28 August rain days and a mean timing of breeding of 14 October; driest springs had 12 rain days and a mean timing of 5 October; overall mean timing was 9 October. Rain days had >0·85 mm rainfall, as per Senapathi *et al*. (2010). Estimates of breeding success were generated from parameters in Table 1.

Of the 1581 eggs laid in first clutches within the study period, 722 survived to fledge. Agriculture had an adverse effect on breeding success in a simple GLMM ($\chi_{1}^{2}$ = 3·96, *P *=* *0·047) and in an interaction with the timing of breeding ($\chi_{1}^{2}$ = 4·91, *P *=* *0·027). The interactive effect was still apparent in the complex model ($\chi_{1}^{2}$ = 4·28, *P *=* *0·038; Table 1), such that the seasonal decline in breeding success became progressively worse as the area of agriculture around a nest site increased (Fig. 1). This interaction implies that if the relationship between breeding success and agriculture were used in isolation to estimate the demographic impact of agriculture, it would under‐estimate breeding success in dry (early) springs and over‐estimate it in wet (late) springs. We quantified this bias by using the complex model in Table 1 to compare breeding success between wet, average and dry springs as the extent of agriculture increased (Fig. 2). Our analysis showed that the bias is significant, representing up to a 38% under‐estimate in dry springs and up to a 35% over‐estimate in wet springs.

**Table 1:** Complex model of breeding success using binomial errors, a logit link and a female identity random effect (variance component ± SD = 0·431 ± 0·656). Timing refers to the first egg date of the clutch. Male PBE refers to the male parent's prior breeding experience. Cavity type is a two‐level categorical variable (box and natural cavities); values for boxes are as for the intercept

Parameter	Estimate	SE	*z*	*P*
Intercept	0·162	0·741	0·218	0·827
Clutch size	0·084	0·146	0·575	0·565
Male PBE (years)	−0·017	0·038	−0·438	0·661
Cavity type (natural)	−0·154	0·270	−0·571	0·568
Local density	−0·068	0·071	−0·957	0·338
Timing	−0·0005	0·011	−0·044	0·965
December rainfall (cm)	**0·025**	**0·012**	**2·015**	**0·044**
Agriculture (%)	0·016	0·015	1·102	0·270
Timing × December rainfall	**−0·001**	**0·0003**	**−2·953**	**0·003**
Timing × agriculture	**−0·001**	**0·0004**	**−2·038**	**0·042**

Significance values presented are Type III, with significant terms (*P *<* *0·05) highlighted in bold. Tests of individual terms of interest are given in the main text. Model based on 313 breeding attempts, with 130 females.

### Habitat and Prey

**Figure 3:**
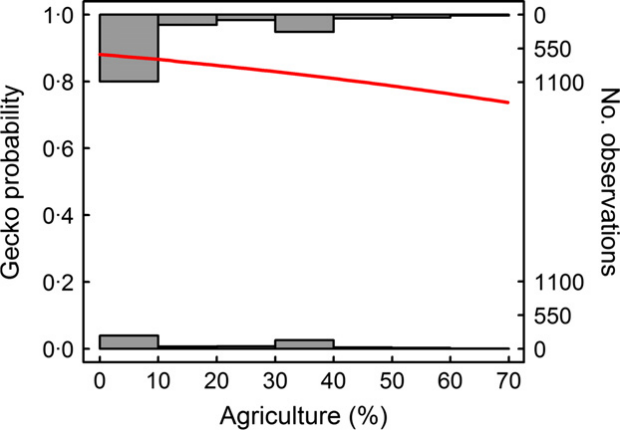
Effect of agriculture on probability of gecko delivery. Histograms represent number of observations when a gecko is delivered (top bars) vs. another prey type (bottom bars). Trend line is based on estimates from the final model in Table 2.

Agriculture reduced the probability of gecko delivery in both the univariate model ($\chi_{1}^{2}$ = 17·3, *P *<* *0·001) and in the complex model containing other background variables (Fig. 3; Table 2). Notably, there was no evidence of any intra‐seasonal pattern (i.e. the date of the prey delivery was not a significant predictor of gecko delivery) or any interaction between the date of delivery and agriculture (Table 2).

**Table 2:** Complex model of gecko delivery probability, using binomial errors, a logit link and kestrel identity as a random effect (variance component ± SD = 1·051 ± 1·025). Season refers to the year that the breeding attempt occurred. Date is the date within the breeding season on which the prey delivery occurred

Parameter	Estimate	SE	*z*	*P*
Intercept	**1·858**	**0·565**	**3·287**	**0·001**
Season	**−0·056**	**0·021**	**−2·691**	**0·007**
Age (years)	−0·051	0·042	−1·204	0·228
Elevation (m)	0·0001	0·001	0·071	0·943
December rainfall (cm)	−0·001	0·005	−0·280	0·779
Local pair density	0·174	0·093	1·875	0·061
Agriculture (%)	**−0·048**	**0·019**	**−2·456**	**0·014**
Sex (male)	**0·753**	**0·328**	**2·297**	**0·022**
Date	0·00002	0·003	0·007	0·994
Agriculture × sex (male)	0·020	0·016	1·284	0·199
Agriculture × date	0·0002	0·0001	1·304	0·192

Significance values presented are Type III, with significant terms (*P *<* *0·05) highlighted in bold. Model based on 1788 records, with 238 kestrels.

### Prey and Breeding Success

**Figure 4:**
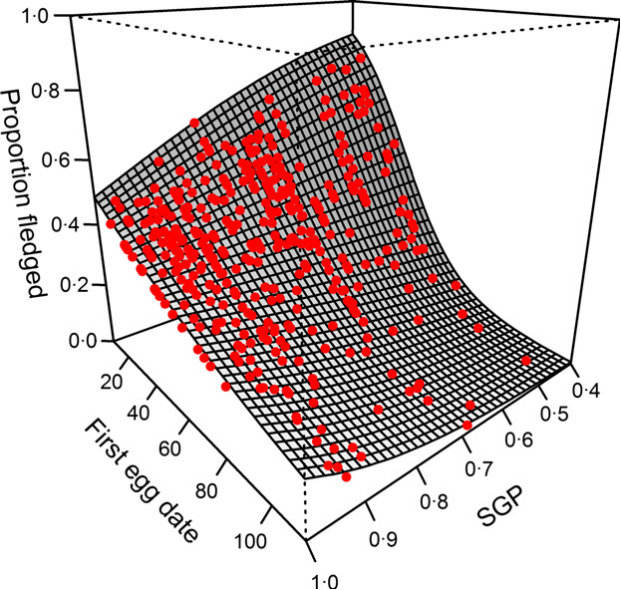
Interactive effect of timing of breeding (first egg date) and site‐specific gecko delivery probability (SGP) on breeding success. Surface shows predicted trend from parameters in Table S4 (Supporting information). Points show combinations of SGP and timing in raw data, with model predicted breeding success. First egg date scale is from 1 (1 September) to 113 (22 December). Graph made using the lattice package (Sarkar 2008).

If variation in the prey community were sufficient to explain the effect of agriculture upon breeding success, then we would expect to see that site‐specific differences in the prey community relate directly to breeding success in a similar way to agriculture. The interaction between site‐specific probability of gecko delivery (SGP) and the timing of breeding had a significant effect on breeding success in the complex model ($\chi_{1}^{2}$ = 7·85, *P *=* *0·005; Table S4, Supporting information), in the form of progressively lower success with declining SGP and later breeding (Fig. 4). When the agriculture and timing interaction was added to this model, the resulting model, containing both agriculture and SGP, was significantly better at explaining variation in breeding success ($\chi_{2}^{2}$ = 9·96, *P *=* *0·007) than the model containing only SGP (i.e. SGP did not explain all of the agriculture effect on breeding success).

## Discussion

Our results suggest that agriculture modifies the seasonal decline in breeding success in our study population; the rate of this decline becoming more rapid as the area of agriculture around a nest site increases. Our results also suggest that this relationship may be at least partly due to spatial variation in native, arboreal geckos.

Our interpretation of these patterns and their implications for our understanding of the demographic impacts of environmental change rest on the validity of our statistical analysis. Since our analysis is correlative, it is possible that the patterns we attribute to agriculture are actually the result of an unmeasured variable correlated with agriculture and breeding success. Although we cannot completely exclude this possibility, there are three reasons we consider it unlikely. First, the interaction between agriculture and timing of breeding is evident in both simple and complex models. The complex model includes variables known to affect breeding success in our study population based on extensive work. This reduces the likelihood that agriculture is simply a surrogate for another variable that affects breeding success. Secondly, our results are robust to alternative random effect structures and the modelling of breeding success as nest‐scale success vs. failure (Tables S1 and S2, Supporting information). This suggests that the interaction between timing of breeding and agriculture is important in explaining complete nest failure, which is reinforced by the fact that we were unable to detect a similar effect when breeding success was modelled excluding breeding attempts that failed completely (Table S3, Supporting information).

Thirdly, our separate analysis of prey delivered to nests provides a mechanistic explanation for the patterns involving agriculture. We show that the likelihood of native, arboreal *Phelsuma* geckos being delivered to nests decreases as the area of agriculture increases. Gecko abundance is likely to be lower in agriculture because sugar cane lacks the forage, shelter and egg‐laying sites provided by tree cover (Harmon, Harmon & Jones 2007) and overall prey availability to kestrels may be limited by a lack of perches usually provided by emergent trees and forest canopy that enable Mauritius kestrels to hunt effectively (Burgess *et al*. 2009). It has been shown elsewhere that the seasonal decline in breeding success occurs mainly because late nests are more likely to experience rainfall during the nestling period (Senapathi *et al*. 2011). Rainfall at this time can cause direct nestling mortality due to flooding or chilling and indirect mortality by reducing the hunting efficiency of parents provisioning chicks. The combined effects of agriculture on prey availability and late season rainfall on hunting efficiency may at least partly explain the interaction we found between agriculture and timing of breeding on breeding success. Prey availability is only part of the explanation because our analysis also shows that the patterns of prey delivery alone were insufficient to account for the interaction. This is perhaps unsurprising since prey delivery records do not assess rates of prey, energy or nutrient delivery to nests, which are also likely to be important. Taken together, this evidence suggests that the patterns we report are likely to be ecologically important rather than statistical artefacts.

How might prey availability affect breeding success? One explanation is that Mauritius kestrel chicks are adapted to consume native geckos. Compared with adults, chicks are less able to cast a pellet containing the roughage found in non‐native prey, such as mammal fur and agama lizard scales. These indigestible remains can cause compaction of the proventriculus, a fatal condition noted in captive‐bred and wild chicks (Jones *et al*. 1995). Field visits to nests often reveal chicks with the indigestible tails of agama lizards protruding from their bills, and it is common to find the uneaten remains of Asian musk shrew*,* agama lizard and small birds in nests containing chicks at a range of ages (S.J. Cartwright, personal observation). This suggests that geckos may represent the optimal food for nestlings, although kestrels will provide alternative, potentially inappropriate food if gecko availability is low. Changes in the ratio of food types in the diet may thus affect the survival of nestlings. Additionally, since prey availability did not entirely explain the effect of agriculture on breeding success, an array of factors probably link agriculture to reduced breeding success, of which the change in the prey community is one key part. On‐going conservation of this kestrel population would benefit from research into the rate of prey deliveries across habitats to better understand the role of a changing prey community in agriculture's effect on breeding success.

What are the wider implications of our results for how we assess and manage the impacts of environmental change on demography and hence population dynamics? There is a considerable body of ecological knowledge concerning the impacts of agriculture (e.g. Newton 2004; Wilson, Evans & Grice 2009; and examples therein) and climate change (e.g. Robinson, Baillie & Crick 2007; Møller, Fiedler & Berthold 2010; and examples therein) on demography, but most of this work considers these drivers in isolation. In contrast, most wild populations are exposed to multiple, concurrent changes (Sala *et al*. 2000), particularly the combined effects of habitat and climate change (Mantyka‐Pringle, Martin & Rhodes 2012). We show that quantifying the relationship between breeding success and agriculture in isolation (i.e. under average rainfall conditions) under‐estimates breeding success in dry (early) springs and over‐estimates breeding success in wet (late) springs (Fig. 2). This bias becomes more acute as the extent of agriculture increases. Since springs are getting wetter in our study area (Senapathi *et al*. 2010), our results suggest that the demographic impact of agriculture has worsened over time; a process that would have remained undetected if the relationship between agriculture and breeding success had been explored in isolation from climate effects. This highlights an important general point that climate change could in future modify the demographic impact of historical land‐use change so such relationships should be regarded as dynamic rather than static.

Although we need to explore the implications of our results for population growth and persistence in more detail, they do have some direct implications for management. First, large‐scale forest restoration is an obvious response to the negative impacts of agriculture on this population. An opportunity for such a programme may well arise in future with global changes in the sugar market expected to release 70 km^2^ of marginal land from sugar cane cultivation, with at least 50 km^2^ of this earmarked for forestry (Forestry Service 2007). Although forestry may not equate to restoration of native forest, our results suggest that reducing agricultural habitat *per se* in the buffer around the kestrel population could improve breeding success in affected territories. Given the steep decline in breeding success with relatively modest increases in agriculture cover, any attempt to actively expand the kestrel population should consider large‐scale reforestation that minimizes agriculture within new breeding territories. Secondly, our results suggest that forest restoration would also act as an adaptation strategy to climate change (i.e. wetter springs) because reducing agriculture also reduces the variation in breeding success due to the timing of breeding, particularly the poorer breeding success associated with wet springs (Fig. 2). This illustrates how a more integrated approach to studying the impacts of environmental change can also help identify strategies that simultaneously address the negative impacts of multiple drivers. Since such an approach is inevitably data demanding, it highlights the importance of long‐term population studies in helping us understand the synergies between spatio‐temporal environmental drivers that are a key future concern in conservation biology (Brook, Sodhi & Bradshaw 2008).

## Supporting information


**Fig. S1.** Variation in Mauritius kestrel timing of breeding.
**Table S1.** Models of breeding success with additional random effect structures.
**Table S2.** Additional models of nest‐scale success.
**Table S3.** Additional models of breeding success (excluding failed breeding attempts).
**Table S4.** Model of breeding success with site‐specific gecko probability.
