# Supplementary material for: Agriculture modifies the seasonal decline of breeding success in a tropical wild bird population
Source: J Appl Ecol. 2014 Jul 25;51(5):1387–95. doi: 10.1111/1365-2664.12310 (PMC4279951; doi:10.1111/1365-2664.12310)

Notes on the Supporting Information:

For Tables S1-4 results presented are from generalised linear mixed-effects models (GLMM) with binomial errors and a logit link. Significance values presented are Type III, with significant terms ($P<0.05$) highlighted in bold. Timing refers to the first egg date of the clutch. Male PBE refers to the male parent's prior breeding experience. Cavity type is a two-level categorical variable (box and natural cavities), with values for boxes indicated by the intercept.

In Tables S1-3 results presented are for models with different random effects structures. We compare three different structures in our analyses: a female identity random intercept; female identity and breeding season random intercepts (to account for parent-level and within-breeding season replication of conditions; and breeding territory and breeding season random intercepts (to account for repeated breeding attempts at the same breeding territory and within the same breeding season).

Table S1. Models of breeding success with (1) female identity and breeding season as random effects (variance components ± s.d.: female = 0.432 ± 0.657; season = 0.016 ± 0.125) and (2) breeding territory and breeding season as random effects (variance components ± s.d.: territory = 0.091 ± 0.302; season = 0.022 ± 0.148). Interaction between timing of breeding and agriculture has a significant effect on breeding success (1: $\chi^2_1 = 4.47$, P = 0.034; 2: $\chi^2_1 = 5.85$, P = 0.016). Models based on 313 breeding attempts, with 130 females, over 20 years and 63 territories

	1				2			
Parameter	Estimate	SE	z	P	Estimate	SE	z	P
Intercept	0.163	0.749	0.217	0.828	0.205	0.663	0.309	0.757
Clutch size	0.077	0.147	0.522	0.602	0.009	0.130	0.073	0.942
Male PBE (years)	-0.016	0.038	-0.423	0.673	-0.011	0.034	-0.317	0.751
Cavity type (natural)	-0.160	0.272	-0.588	0.557	-0.258	0.242	-1.066	0.287
Local density	-0.064	0.072	-0.895	0.371	-0.043	0.069	-0.622	0.534
Timing	-0.0002	0.011	-0.019	0.984	0.0025	0.010	0.257	0.797
**December rainfall (cm)**	**0.026**	**0.013**	**2.009**	**0.045**	**0.027**	**0.012**	**2.285**	**0.022**
Agriculture (%)	0.017	0.015	1.171	0.242	0.021	0.013	1.651	0.099
**Timing x December rainfall**	**-0.001**	**0.0003**	**-2.945**	**0.003**	**-0.001**	**0.0003**	**-3.135**	**0.002**
**Timing x agriculture**	**-0.001**	**0.0004**	**-2.081**	**0.037**	**-0.001**	**0.0003**	**-2.426**	**0.015**

Table S2. Models of nest-scale success (defined as a binary response; successful versus failed nest; where a successful nest produces at least one fledgling), with (1) a female identity random effect (variance component ± s.d. = 0.288 ± 0.537); (2) female identity and breeding season as random effects (variance components ± s.d.: female = 0.287 ± 0.536; season = <0.001 ± 0.003) and (3) breeding territory and breeding season as random effects (variance components ± s.d.: territory = 0.043 ± 0.207; season = <0.001 ± <0.001). Interaction between timing of breeding and agriculture has a significant effect on breeding success (1: $\chi^2_1 = 4.86$, P = 0.027; 2: $\chi^2_1 = 4.86$, P = 0.027; 3: $\chi^2_1 = 4.80$, P = 0.029). Models based on 313 breeding attempts, with 130 females over 20 seasons and 63 territories

	1				2				3			
Parameter	Estimate	SE	z	P	Estimate	SE	z	P	Estimate	SE	z	P
Intercept	-0.228	1.359	-0.168	0.867	-0.227	1.359	-0.167	0.867	-0.060	1.289	-0.046	0.963
**Clutch size**	**0.807**	**0.264**	**3.056**	**0.002**	**0.808**	**0.264**	**3.058**	**0.002**	**0.731**	**0.255**	**2.862**	**0.004**
Male PBE (years)	-0.005	0.073	-0.069	0.945	-0.005	0.073	-0.069	0.945	-0.002	0.071	-0.022	0.983
Cavity type (natural)	0.401	0.503	0.797	0.425	0.401	0.503	0.798	0.425	0.362	0.484	0.748	0.455
Local density	-0.237	0.132	-1.800	0.072	-0.237	0.132	-1.800	0.072	-0.228	0.126	-1.802	0.072
Timing	-0.011	0.021	-0.524	0.600	-0.011	0.021	-0.529	0.597	-0.011	0.019	-0.575	0.565
December rainfall (cm)	0.008	0.026	0.323	0.747	0.008	0.026	0.319	0.750	0.008	0.024	0.354	0.723
Agriculture (%)	0.050	0.032	1.558	0.119	0.050	0.032	1.559	0.119	0.049	0.030	1.614	0.107
Timing x December rainfall	-0.001	0.001	-1.034	0.301	-0.001	0.001	-1.029	0.303	-0.001	0.001	-1.099	0.272
**Timing x agriculture**	**-0.002**	**0.001**	**-2.075**	**0.038**	**-0.002**	**0.001**	**-2.075**	**0.038**	**-0.001**	**0.001**	**-2.103**	**0.035**

Table S3. Models of breeding success (with 80 breeding attempts that produced no fledglings excluded from analysis), with (1) a female identity random effect (variance component ± s.d. = 0.019 ± 0.138); (2) female identity and breeding season as random effects (variance components ± s.d.: female = 0.006 ± 0.079; season = 0.034 ± 185) and (3) breeding territory and breeding season as random effects (variance components ± s.d.: territory = 0.024 ± 0.155; season = 0.036 ± 0.189). Interaction between timing of breeding and agriculture has no significant effect on breeding success (1: χ^2_1 = 0.78, P = 0.377; 2: χ^2_1 = 1.06, P = 0.302; 3: χ^2_1 = 0.97, P = 0.326). Models based on 233 breeding attempts, with 106 females over 18 seasons and 54 territories

	1				2				3			
Parameter	Estimate	SE.	z	P	Estimate	SE	z	P	Estimate	SE	z	P
**Intercept**	**2.053**	**0.828**	**2.480**	**0.013**	**2.093**	**0.843**	**2.484**	**0.013**	**2.072**	**0.848**	**2.444**	**0.015**
**Clutch size**	**-0.431**	**0.157**	**-2.741**	**0.006**	**-0.456**	**0.160**	**-2.850**	**0.004**	**-0.451**	**0.162**	**-2.779**	**0.005**
Male PBE (years)	-0.014	0.037	-0.375	0.707	-0.013	0.037	-0.342	0.733	-0.015	0.038	-0.383	0.702
**Cavity type (natural)**	-0.450	0.235	-1.915	0.055	**-0.471**	**0.235**	**-2.002**	**0.045**	-0.462	0.244	-1.897	0.058
Local density	0.029	0.067	0.430	0.667	0.034	0.067	0.503	0.615	0.045	0.074	0.608	0.543
Timing	0.006	0.013	0.465	0.642	0.007	0.013	0.568	0.570	0.007	0.013	0.532	0.595
December rainfall (cm)	0.022	0.014	1.598	0.110	0.024	0.015	1.618	0.106	0.023	0.015	1.596	0.111
Agriculture (%)	0.003	0.014	0.242	0.809	0.006	0.014	0.404	0.687	0.006	0.015	0.402	0.688
Timing x December rainfall	-0.001	0.0004	-1.696	0.090	-0.001	0.0004	-1.745	0.081	-0.001	0.0004	-1.731	0.083
Timing x agriculture	-0.0004	0.0004	-0.898	0.369	-0.0004	0.0004	-1.045	0.296	-0.0004	0.0004	-0.992	0.321

Table S4. Model of breeding success with female identity as the random effect (variance component ± s.d. = 0.429 ± 0.655). Test of interaction between timing of breeding and SGP is given in the main text. Model based on 313 breeding attempts, with 130 females

Parameter	Estimate	SE	z	P
**Intercept**	**3.762**	**1.295**	**2.905**	**0.004**
Clutch size	0.022	0.143	0.151	0.880
Male PBE (years)	-0.003	0.038	-0.086	0.931
Cavity type (natural)	-0.196	0.267	-0.735	0.462
Local density	-0.011	0.070	-0.162	0.872
**Timing**	**-0.079**	**0.027**	**-2.913**	**0.004**
**December rainfall (cm)**	**0.025**	**0.013**	**1.977**	**0.048**
**SGP**	**-4.216**	**1.438**	**-2.932**	**0.003**
**Timing x December rainfall**	**-0.001**	**<0.001**	**-2.934**	**0.003**
**Timing x SGP**	**0.090**	**0.033**	**2.686**	**0.007**

Figure S1. Variation in Mauritius kestrel timing of breeding (first egg date; 'FED') with frequency of spring rainfall. Points show raw data. Heavy line indicates slope of difference in population-mean timing for the driest and wettest springs (12 and 28 rain days in August; FED = 35 and 44, respectively; slope ± SE: 0.585 ± 0.185, $\chi^2_1 = 9.796$, P = 0.002). Dashed line marks population mean timing for wettest spring. First egg date scale is from 1st Sept.

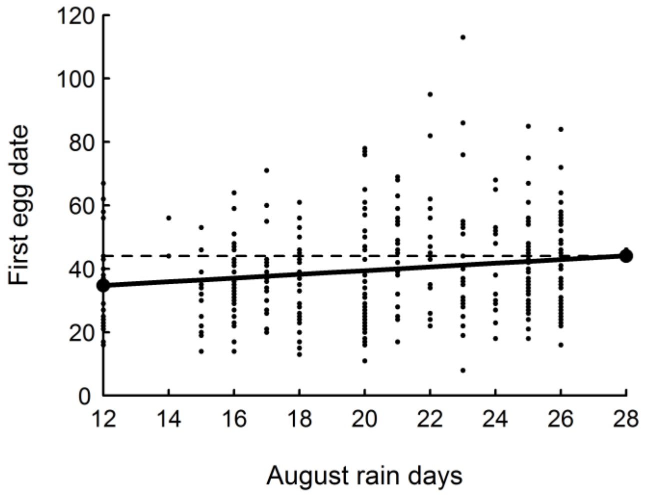

Supplement: Supplementary file 1 — Fig. S1. Variation in Mauritius kestrel timing of breeding. Table S1. Models of breeding success with additional random effect structures. Table S2. Additional models of nest‐scale success. Table S3. Additional models of breeding success (excluding failed breeding attempts). Table S4. Model of breeding success with site‐specific gecko probability. [file JPE-51-1387-s001.pdf]
